# Prof. W. F. Westmoreland’s New Instrument

**Published:** 1860-05

**Authors:** 


**Prof. W. F. Westmoreland's New Instrument.**

At the recent meeting of the Medical Association of Georgia, Dr. W. F. Westmoreland, Prof. of Surgery in the Atlanta Medical College, presented a new *Urethrotome*, of his own invention, intended specially for the section of strictures of the urethra, without the necessity of dilatation. There are many cases of this character, which prove exceedingly difficult to manage, and indeed are among the most annoying to the sufferer, as well as to the Surgeon. With this instrument, the section can be successfully and safely made in cases where the number 1 bougie cannot be passed. The advantage claimed for this instrument, in its construction, is a small director, which may be introduced through any stricture, that is not absolutely impenetrable. This instrument has already been practically and successfully tested in Prof. Westmoreland's hands, and a report of cases and cut of the instrument will shortly appear in this Journal. In the meantime, should any gentleman desire to obtain the instrument it can be had of Tiemann, New York City, the manufacturer.

